# Genetic and molecular biology of bladder cancer among Iranian patients

**DOI:** 10.1002/mgg3.1233

**Published:** 2020-04-06

**Authors:** Majid Mojarrad, Meysam Moghbeli

**Affiliations:** ^1^ Department of Medical Genetics and Molecular Medicine School of Medicine Mashhad University of Medical Sciences Mashhad Iran

**Keywords:** bladder cancer, genetic, Iran, panel marker, risk factor

## Abstract

**Background:**

Bladder cancer (BC) is the sixth common cancer among Iranians. Various risk factors such as smoking, body mass index, chronic infection, age, and genetic factors are associated with BC progression.

**Methods:**

It has been shown that a significant ratio of patients have tumors with muscle bladder layer invasion and poor prognosis at the time of diagnosis. Therefore, the early detection of tumors is required to reduce the mortality rate of BC cases. Since there is a wide geographical incidence variation in BC in Iran, it seems that the ethnic and genetic factors can be the main risk factors among Iranian BC patients.

**Results:**

For the first time, in present review we have summarized all of the reported genes among Iranian BC patients until now which were significantly associated with tumorigenesis. Moreover, we categorized all of the reported genes based on their cell and molecular functions to clarify the genetic and molecular biology of BC among Iranian population.

**Conclusion:**

This review paves the way of determination of a population‐based genetic panel markers for the early detection of BC in this population.

## Graphical abstract

In present review we have summarized all of the reported genes among Iranian BC patients until now which were significantly associated with tumorigenesis. We categorized all of the reported genes based on their cell and molecular functions to clarify the genetic and molecular biology of BC among Iranian population. This review paves the way of determination of a population based genetic panel markers for the early detection of BC in this population.

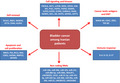

## 1 INTRODUCTION

**FIGURE 1:**
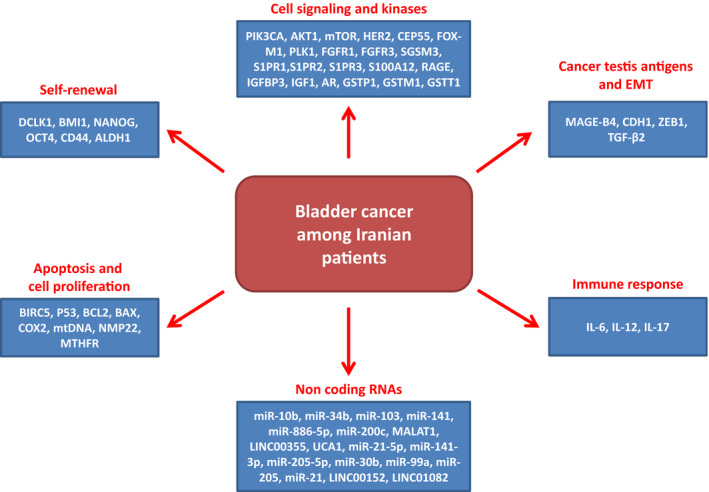
Cell and molecular processes which are involved in bladder cancer progression among Iranian patients

Cancer is the third cause of deaths among Iranian population (Saadat et al., 2015). Almost 90,000 new cancer cases are diagnosed per year which is estimated to be doubled by the 2020 in this population. There is also different incidence of cancer in males and females in which more than 55% of patients were observed among Iranian males (Rafiemanesh et al., 2015). Bladder cancer (BC) is the 14th common cause of cancer‐related deaths globally (Ferlay et al., 2015) and is the 6th most common malignancy among Iranians (Jafari‐Koshki, Arsang‐Jang, & Mahaki, 2017). BC is histologically categorized in three subtypes including transitional cell carcinoma (TCC), squamous cell carcinoma (SCC), and adenocarcinoma. Although TCC is the most common type of bladder cancer (90%) in developed countries, SCC is the most frequent type (75%) in developing countries (Pakzad et al., 2015). In Iran, bladder and papillary TCC include 43.89% and 49.86% of cases, respectively (Rafiemanesh, Lotfi, Bakhtazad, Ghoncheh, & Salehiniya, 2018). There is clear correlations between BC and several risk factors such as smoking, body mass index, chronic infections, and genetic factors (Afshari, Janbabaei, Bahrami, & Moosazadeh, 2017; Freedman, Silverman, Hollenbeck, Schatzkin, & Abnet, 2011; Jemal et al., 2011). Smoking, age, analgesic use, and hair dye were also the risk factors among Iranian cases (Ramezani, Naderi, Almasi, & Sadeghi, 2016; Shakhssalim et al., 2010). The highest BC incidences are observed in developed and some African countries, whereas the Middle East and North Africa have the highest mortality rates (Chavan, Bray, Lortet‐Tieulent, Goodman, & Jemal, 2014). BC is more common (about 4 times) among men compared with women that can be associated with higher smoking behaviors among men (Murta‐Nascimento et al., 2007). The mortality rate of BC is also higher among men compared with women (Ahmadi et al., 2012). Recent reports have shown that there is a rising trend of BC incidence among Iranian population in which the total ASIR increased from 10.47 to 18.2 per 100,000 in both genders (Rafiemanesh et al., 2018). The standardized incidence rate is reported between 26.9 and 5.3 per 100,000 among European males and females, respectively (Ferlay et al., 2013). Whereas the rates are 14.42 and 3.78 among Iranian men and women, respectively, which are lower than European countries (Rafiemanesh et al., 2018). There is also a geographical variation in BC incidence in Iran in which the Yazd, Kurdistan, Gilan, and Fars provinces have the highest, whereas the Hormozgan, Hamedan, and Sistan‐Baluchestan provinces had the lowest rates (Esmaeimzadeh, Salahi‐Moghaddam, & Khoshdel, 2015). About 20% of primary BC tumors have muscle bladder layer invasion and poor prognosis at the time of diagnosis. And there is a high ratio of tumor relapse after tumor resection (Goodison, Rosser, & Urquidi, 2013). Cystoscopy and urine cytology are the common diagnostic methods of BC (Goodison et al., 2013; Griffiths & Action on Bladder Cancer, 2013). However, small papillary tumors could be missed by standard white‐light cystoscopy (WLC) which needs fluorescence and narrow‐band imaging (NBI) cystoscopy (Cheung, Sahai, Billia, Dasgupta, & Khan, 2013). Therefore, regarding the different incidence ratios between different countries and areas, it is required to introduce population‐based novel diagnostic and screening methods for the early detection of BC and high‐risk cases. In present review we have summarized all of the reported genes among Iranian BC patients until now which were significantly associated with tumorigenesis (Table. 1). Moreover, for the first time we categorized all of the reported genes based on their cell and molecular functions (Figure 1) to clarify the genetic and molecular biology of BC among Iranian population.

**TABLE 1:** All of the involved genes in bladder cancer susceptibility among Iranian patients

study (et al)	Year	Gene	Sample	Results
Atlasi	2009	BIRC5	30 Tumors 28 Controls	Overexpression
Mowla	2005	BIRC5	17 Patients	Overexpression
Nouraee	2009	BIRC5	30 Patients	Overexpression
Kalantari	2007	P53	50 Patients 10 Controls	Overexpression in papillary low‐grade TCC
Bazrafshani	2016	P53	60 Patients	Mutation
Golestani Eimani	2014	BCL2, BAX	40 Patients	BCL2/BAX expression ratio as prognostic marker
Tabriz	2013	COX2	92 Patients	Correlation with age, grade, and lymph node
Shakhssalim	2013	mtDNA	26 N/T^a^ 504 Controls	Polymorphism was correlated with BC risk
Jamshidian	2008	NMP22	76 Patients 75 Controls	Higher urine levels
Safarinejad	2011	MTHFR	158 Patients 316 Controls	Polymorphism was correlated with BC risk
Shafiei	2019	DCLK1	472 Patients	Overexpression
Shafaroudi	2008	BMI1	40 Patients 8 Controls	Overexpression
Amini	2014	BMI, NANOG	10 Patients	Overexpression
Atlasi	2007	OCT4	32 Tumors 22 Normal	Overexpression
Hatefi	2012	OCT4	30 Patients	Correlation with grade and stage
Keymoosi	2014	ALDH1A1, CD44	159 Patients	ALDH1A1 was correlated with grade, stage, and age
Nekoohesh	2018	miR‐10b, miR‐34b, miR‐103, miR‐141	66 Patients 53 Controls	miR‐10b, miR‐34b, and miR‐103 overexpressions miR‐141 underexpression
Khoshnevisan	2015	miR‐886‐5p	70 Patients	miR‐886‐5p overexpression in high‐grade
Mahdavinezhad	2015	miR‐200c, miR‐30b, miR‐141	35 N/T	Overexpression
Ghorbanmehr	2019	miR‐21‐5p, miR‐141‐3p, miR‐205‐5p	45 Patients 22 Controls	miR‐21‐5p, miR‐141‐3p, and miR‐205‐5p urinary overexpressions
Mahdavinezhad	2015	miR‐30b, miR‐141, miR‐200c	35 N/T	Muscle‐invasive BC had lower levels of expression
Ganji	2017	miR‐99a, miR‐205	36 N/T	miR‐99a and miR‐205 underexpressions
Monfared	2013	TGF‐β, miR‐21	30 N/T	TGF‐β underexpression mainly in low‐grade BC miR‐21 overexpression in high‐grade BC
Ousati Ashtiani	2017	LINC00152, LINC01082	50 N/T	LINC00152 and LINC01082 underexpressions
Yazarlou	2018	MALAT1, LINC00355, UCA1‐203	59 Patients 24 Controls	MALAT1, LINC00355, and UCA1‐203 overexpressions in urinary exosomes
Bizhani	2018	PIK3CA, AKT1, mTOR	235 Patients 254 Controls	Polymorphisms were correlated with BC risk
Ousati Ashtiani	2018	PIK3CA	50 N/T	Mutation
Jalali Nadoushan	2007	HER‐2/neu	75 Patients	Overexpression in higher grade of TCC
Seyedabadi	2018	CEP55, FOXM1, PLK1	36 Patients	CEP55, FOXM1, and PLK1 overexpressions
Ousati Ashtiani	2017	FGFR1, FGFR3	50 N/T	FGFR1 and FGFR3 overexpressions
Hashemi	2018	SGSM3	143 Patients 144 Controls	Polymorphisms were correlated with BC risk
Palangi	2019	S1PR1, S1PR2, S1PR3	41 Patients 26 Controls	Overexpressions
Khorramdelazad	2015	S100A12, RAGE	17 Patients	Overexpressions
Safarinejad	2011	IGFBP‐3	162 Patients 324 Controls	Polymorphisms were correlated with BC risk
Mashhadi	2014	AR	120 Patients 132 Controls	Correlation with grade and stage
Safarinejad	2013	GSTM1, GSTT1, GSTP1	166 Patients 332 Controls	Polymorphisms were correlated with BC risk
Yazarlou	2018	MAGE‐B4	59 Patients 24 Controls	Overexpression
Khorrami	2012	CDH1	180 Patients	Was correlated with recurrent BC
Mahdavinezhad	2017	ZEB1	35 Patients	Overexpression
Ebadi	2014	IL‐12, IL‐6	261 Patients 251 Controls	Polymorphisms were correlated with BC risk
Doroudchi	2013	IL‐17A	201 Patients 59 Controls	Overexpression
Baharlou	2014	IL‐17, TGF‐β	40 Patients 38 Controls	Decreased serum levels
Baharlou	2015	IL‐17, TGF‐β	37 Patients	IL‐17 decreased TGF‐β expressions in early stages

^a^ Tumor tissues and normal margins.

## 2 APOPTOSIS AND CELL PROLIFERATION

Apoptosis is a programmed cell death involved in regulation of cell proliferation following DNA damage. Therefore, apoptosis aberration can be associated with tumor progression. Apoptosis is regulated by two protein families including *BCL‐2* and *IAP*. Survivin (*BIRC5*) (OMIM: 603352) belonged to the inhibitors of apoptosis (IAPs) family and is involved in regulation of apoptosis and cell proliferation (Ambrosini, Adida, & Altieri, 1997). Survivin is mainly expressed in tumor cells, with a rare expression in normal differentiated cells. It has been shown that the full‐length survivin and survivin‐2B isoforms were the highest and lowest expressed isoforms in bladder tissues. Moreover, the normal tissues had low levels of survivin and survivin‐DEx3 expressions. There was a significant association between the levels of survivin/survivin‐DEx3 expressions and tumor aggressiveness among a sample of Iranian BC patients (Atlasi, Mowla, & Ziaee, 2009). Another study has been reported that the recurrent bladder tumors had higher survivin and/or survivin‐∆Ex3 expression compared with primarily tumors among a subpopulation of Iranian patients (Mowla, Emadi Bayegi, Ziaee, & Nikpoor, 2005). The survivin and survivin‐∆Ex3 overexpressions resulted in declined 5 years survival rate among a sample of Iranian subjects. Moreover, the overexpression of survivin and survivin‐∆Ex3 was more frequent among high‐grade and stage tumors. They also observed that the survivin‐2B overexpression was correlated with decreased risk of mortality. Therefore, they introduced the survivin and survivin‐∆Ex3 isoforms as markers of aggressive and recurrence BC, whereas the surviving‐2B isoform was protective (Nouraee et al., 2009). P53 is a tumor suppressor involving in cell‐cycle regulation and apoptosis (Esrig et al., 1994; Lane, 1992). It has been shown that there was significant difference in *P53* (OMIM: 191170) expression between papillary urothelial neoplasm of low malignant potential (PUNLMP) and papillary low‐grade TCC in which the P53 overexpression was more frequent among papillary low‐grade TCC tumors (Kalantari & Ahmadnia, 2007). A mutational analysis of TP53 was performed among a group of Iranian BC patients which showed that a noticeable numbers of patients were carriers of deletion/duplication mutations. Majority of duplication and deletion changes were observed in exons 6 and 1 (Bazrafshani et al., 2016). Apoptosis is a critical cellular response toward genotoxic stress which sensitizes the tumors cells to chemotherapeutic drugs (Debatin, 2004). The *BCL2* (OMIM: 151430) and *BAX* (OMIM: 600040) are anti‐ and proapoptotic regulators, respectively. It has been observed that the patients with shorter relapse‐free time had high BCL2/BAX ratio expression in a sample of Iranian BC cases which introduced BCL2/BAX expression ratio as a significant prognostic marker of low‐grade BC (Golestani Eimani et al., 2014). Cyclooxygenases (COXs) are a family of myeloperoxidases catalyzing the prostaglandin synthesis from arachidonic acid (Chandrasekharan & Simmons, 2004). *COX‐2* (OMIM: 600262) is a critical factor during prostaglandins production and is involved in tumorigenesis through apoptosis inhibition, angiogenesis, and metastasis induction (Meric et al., 2006; Pruthi, Derksen, Gaston, & Wallen, 2004). *COX‐2* downregulates proapoptotic NO in tumor cells following the prostaglandin production (Cao & Prescott, 2002). It activates the *PPAR* through prostaglandin I2 (Breyer, Bagdassarian, Myers, & Breyer, 2001). The *COX‐2* inhibition results in impaired *PPARδ* activation which activates BAD proapoptotic factor (Liou, Ghelani, Yeh, & Wu, 2007). It has been shown that there were correlations among *COX‐2* expression, age, grade, and lymph node involvement among a subpopulation of Iranian BC patients. The high‐grade tumors had higher levels of *COX‐2* expression compared with other grades (Tabriz, Olfati, Ahmadi, & Yusefnia, 2013). Mitochondria have a critical role in apoptosis regulation through modulation of Ca^2+^ signaling in which the mitochondrial Ca^2+^ accumulation leads to apoptosis. Mitochondrial DNA (mtDNA) contains several genes such as electron transport chain subunits, tRNAs, rRNAs, and a noncoding sequence (D‐loop) (Suzuki et al., 2003). The D‐loop regulates replication and transcription of mtDNA (Yu et al., 2007). Mitochondrial dysfunction is associated with various degenerative and metabolic disorders and cancer. The germ line and somatic mtDNA mutations are linked with mitochondrial disorders and cancer, respectively. Regarding the lack of protective histones and DNA repair processes, mtDNA is more prone for the mutation accumulation compared with nuclear DNA. Mutational analysis of D‐Loop sequences was performed among a subpopulation of Iranian BC patients compared with their corresponding normal margins. It has been shown that there was a significant correlation between D‐loop C16069T polymorphism and BC (Shakhssalim et al., 2013). Sustained proliferation is one of the hallmarks of cancer that can be observed by deregulation of cell growth and DNA replication. A normal DNA replication is required to maintain the genomic stability. Nuclear matrix proteins (NMPs) are nucleus structural proteins involved in DNA replication and gene expression (Pardoll, Vogelstein, & Coffey, 1980). It has been observed that the BC patients had higher levels of urine *NMP22* compared with controls. Moreover, there were significant correlations between the urine levels of *NMP22* and stage and grade. Therefore, they introduced urine *NMP22* as a noninvasive and sensitive diagnostic method for BC among a subpopulation of Iranian cases (Jamshidian, Kor, & Djalali, 2008). Smoking is one of the main risk factors of BC. Reactive free radicals are the most important smoking‐related carcinogens. Therefore, it is important for the smokers to have high intakes of antioxidants such as folate and B vitamins (B12, B6, and B2). *MTHFR* (OMIM: 607093) is involved in folate and vitamin B12 metabolisms and methionine production. Folate is a critical coenzyme during DNA synthesis and repair. It has been observed that there was an association between 1298AC and 1298CC genotypes of *MTHFR* and increased risk of BC among a subpopulation of Iranian patients. The 1298AA genotype significantly reduced BC risk. Moreover, the 677CT and 677CC genotypes were correlated with tumor aggressiveness. They observed that the 677CT‐1298AC and 677CC‐1298CC carriers had the highest BC susceptibility in this population (Safarinejad, Shafiei, & Safarinejad, 2011a).

## 3 SELF‐RENEWAL

Cancer stem cells (CSCs) are a subpopulation of tumor cells with self‐renewal and drug resistance characteristics which are associated with tumor progression and metastasis (Moghbeli, Moghbeli, Forghanifard, & Abbaszadegan, 2014; Moghbeli et al., 2019). There are various cell surface markers for detection and isolation of CSCs in BC (Amini, Fathi, Mobalegi, Sofimajidpour, & Ghadimi, 2014; Sedaghat et al., 2017). Doublecortin‐like kinase 1 (*DCLK1*) (OMIM: 604742) as a serine/threonine protein kinase is a specific CSC marker in solid tumors associated with apoptosis, cell proliferation, and epithelial–mesenchymal transition (EMT) (Bailey et al., 2014; Fan, Qian, & Dai, 2017; Ikezono et al., 2017). It has been reported that there was increased *DCLK1* expression among a sample of Iranian BC cases in comparison with normal margins. Moreover, the *DCLK1* overexpression was correlated with higher stage, grade, poor prognosis, low survival rate, and tumor aggressiveness (Shafiei et al., 2019). *BMI1* (OMIM: 164831) is belonged to the polycomb group (PcG) proteins which are transcriptional suppressors (Orlando, 2003). Moreover, *BMI1* is also critical for the self‐renewal of normal and cancer stem cells (Molofsky et al., 2003) which can be associated with its inhibitory function on INK4A/ARF locus. It has been reported that the BC tumor tissues had higher levels of *BMI1* expression compared with normal margins. Moreover, there was a significant correlation between *BMI1* expression and stage in which stage Ta tumors had significantly lower levels of *BMI1* compared with tumors with stages T1 and T2 among a subpopulation of Iranian BC cases (Shafaroudi et al., 2008). Self‐renewal and pluripotency are the main features of normal and CSCs during embryogenesis and tumorigenesis. *OCT4* (OMIM: 164177), *SOX2* (OMIM: 184429), and *NANOG* (OMIM: 607937) are important transcription factors during embryogenesis and differentiation (Chakravarthy et al., 2008; Lee, Kim, Rho, Han, & Kim, 2006; Pan & Thomson, 2007). *BMI* is also a transcriptional repressor associated with hematopoiesis and senescence (Leung et al., 2004). It has been observed that the majority of BC cases had *NANOG* and *BMI* overexpressions among a subpopulation of Iranian patients (Amini et al., 2014). *OCT4* is an essential transcription factor for the self‐renewal maintenance in pluripotent cells, somatic, and cancer stem cells. Therefore, *OCT4* is acutely downregulated after the differentiation of stem cells (Pardo et al., 2010; van den Berg et al., 2010). It has been observed that there was *OCT4* overexpression in a sample of Iranian BC subjects (Atlasi, Mowla, Ziaee, & Bahrami, 2007). Another study has also been reported that there was a significant association among *OCT4* expression, grade, and tumor stage among a group of Iranian BC patients. There was also a correlation between *OCT4* expression and BC tumor aggressiveness (Hatefi, Nouraee, Parvin, Ziaee, & Mowla, 2012). *CD44* (OMIM: 107269) and aldehyde dehydrogenase 1 (*ALDH1*) (OMIM: 100640) have been used to detect urothelial carcinoma stem cells (Ho, Kurtova, & Chan, 2012; Immervoll, Hoem, Steffensen, Miletic, & Molven, 2011). *ALDH1A1* belonged to the *ALDH1* family and expressed in cancer stem cells (Ginestier et al., 2007). *CD44* is a hyaluronic acid receptor expressed in normal and cancer cells (Jaggupilli & Elkord, 2012). It has been reported that there were significant correlations between *ALDH1A1* overexpression, age, high‐grade and stage tumors, and tumor relapse among a sample of Iranian BC patients. Therefore, they introduced *ALDH1A1* as a high‐grade urothelial tumor marker and can be used for the targeted therapy against the CSCs. Moreover, more aggressive treatment was required for the ALDH1+/CD44+ tumors (Keymoosi, Gheytanchi, Asgari, Shariftabrizi, & Madjd, 2014).

## 4 NONCODING RNAS

Both of epigenetic and genetic changes are involved in tumorigenesis. In contrast to the genetic alterations, the epigenetic changes are reversible and regulate gene expression without DNA alterations. Epigenetic changes include DNA methylation, chromatin modifications, and noncoding RNA which are widely associated with different cellular processes. Therefore, aberrant epigenetic processes can result in neoplastic transformation. Micro‐RNAs are noncoding RNAs involving in posttranscriptional regulation through binding with three untranslated regions (Soukup et al., 2017). Regarding the high stability of miRNAs in biofluids they can be considered as valuable noninvasive markers (Mall, Rocke, Durbin‐Johnson, & Weiss, 2013; Yun et al., 2012). The *KLF4* (OMIM: 602253) and *HOXD10* (OMIM: 142984) have been reported as direct targets of *miR‐10b* (OMIM: 611576) in BC (Xiao et al., 2014). *MiR‐34b‐3p* (OMIM: 611374) can be associated with cell‐cycle regulation through *CCND2* (OMIM: 123833) (Tan, Zhang, Zhou, Liu, & Liang, 2019). The apoptosis and cell cycle can be regulated by *miR‐103/107* which promotes PI3K/AKT signaling through *PTEN* targeting (Yu et al., 2018). *MiR‐141* (OMIM: 612093) can be associated with negative regulation of cell growth and migration and apoptosis induction through targeting *RUNX1* (OMIM: 151385) (Xu, Ge, Zhang, & Zhou, 2018). It has been observed that there were *miR‐10b* (OMIM: 611576), *miR‐34b* (OMIM: 611374), and *miR‐103* (OMIM: 613187) overexpressions and *miR‐141* (OMIM: 612093) underexpression in a subpopulation of Iranian BC cases in comparison with controls. Moreover, there was a correlation between grade and levels of *miR‐10b* expression. The *miR‐141* and *miR‐34b* expressions were also associated with addiction and metastasis, respectively. The low‐grade patients had significant higher urine *miR‐10b* in comparison with controls which can be introduced as a noninvasive marker for the early detection of BC (Nekoohesh et al., 2018). The *miR‐886‐5p* is considered as an oncomir through downregulation of *BAX* which results in apoptosis inhibition (Li et al., 2011). It has been observed that there was a significant *miR‐886‐5p* overexpression in high‐grade in comparison with low‐grade BC samples among a subpopulation of Iranian subjects. They also reported significant upregulation of *miR‐886‐5p* in invasive in comparison with noninvasive tumors (Khoshnevisan et al., 2015). Another study has reported that there were increased expressions of *miR200c* (OMIM: 612092), *miR‐30b*, and *miR‐141* levels in a subpopulation of Iranian BC tissues compared with normal margins. Moreover, *miR‐200c* underexpression was correlated with poor prognosis and tumor progression to muscle (Mahdavinezhad, Mousavibahar, et al., 2015). *MiR‐21‐5p* (OMIM: 611020) induces the cell proliferation and migration through *PTEN* regulation (Folini et al., 2010). It is also involved in EMT process through activation of TGFβ and Hedgehog signaling pathways (Bonci et al., 2016). Moreover, *miR‐21‐5p* is a negative regulator of apoptosis by targeting *FASL*, *BAX*, and *MARCKS* (Li, Li, Sha, Sun, & Huang, 2009; Papagiannakopoulos, Shapiro, & Kosik, 2008). *MiR‐141‐3p* induces tumor cell proliferation through *KLF‐9* (OMIM: 602902) suppression (Li et al., 2017). Downregulation of *miR‐205‐5p* (OMIM: 613147) induces apoptosis and suppress cell proliferation through *ZEB2* (OMIM: 605802) increased expression and *ERBB3* (OMIM: 190151) decreased expression (Jiang et al., 2017). It has been shown that there were significant *miR‐21‐5p*, *miR‐141‐3p*, and *miR‐205‐5p* urinary overexpressions in a sample of Iranian BC cases, among them the *miR‐141‐3p* and *miR‐205‐5p* had highest and lowest levels of expression, respectively (Ghorbanmehr et al., 2019). Regarding metastasis to the muscles, BC is categorized into nonmuscle‐invasive BC (NMIBC) and muscle‐invasive BC (MIBC). *MiR‐200c* induces BC invasion by targeting *RECK* (Cheng et al., 2016). The *miR‐141*, *miR‐200c*, and *miR‐30b* overexpressions were observed in majority of a sample of Iranian BC patients. However, MIBC cases had lower levels of *miR‐30b*, *miR‐141*, and *miR‐200c* expressions compared with NMIBC cases. There were a noticeable correlation between *miR‐141* underexpression and muscle invasion. Moreover, they showed a significant converse association between *miR‐141* expression and grade. Expressions of *MiR‐30b* and *miR‐200‐c* were also correlated with tumor size in which the tumors bigger than 10 cm had decreased expressions compared with tumors smaller than that (Mahdavinezhad, Mousavi‐Bahar, et al., 2015). *MiR‐205* is involved in EMT process through regulation of various genes such as *ZEB1*, *VEGF‐A* (OMIM: 192240), and *CDH1* (OMIM: 192090) (Kenney et al., 2011; Wiklund et al., 2011). *MiR‐99a* (OMIM: 614509) is mainly introduced as a tumor suppressor and targets several important factors including *m‐TOR* (OMIM: 601231), *FGFR3* (OMIM: 134934), and *IGF1* (OMIM: 147440) (Fischbach et al., 2015; Wu, Zhou, Pan, Qu, & Zhou, 2015). It has been reported that there were *miR‐99a* and *miR‐205* underexpressions in BC tissues compared with normal margins among a subpopulation of Iranian subjects. High‐grade tumors with muscle invasion had higher levels of *miR‐99a* expression compared with low‐grade tumors which suggesting that as a marker of poor differentiated tumor cells (Ganji et al., 2017). *MiR‐21* has oncogenic roles through inhibition of several tumor suppressors including *PTEN*, *TPM1* (OMIM: 191010), and *MASPIN* (OMIM: 154790) (Asangani et al., 2008; Zhu et al., 2008). The *miR‐21* can be upregulated by *BMP‐4* (OMIM: 112262) and *TGF‐β* (OMIM: 190180) in vascular smooth muscle cells (Davis, Hilyard, Lagna, & Hata, 2008). *TGF‐β* is involved in various processes such as cell growth, differentiation, and apoptosis (Keski‐Oja, Koli, & von Melchner, 2004). It has been observed that there was significant *TGF‐β* underexpression mainly in low‐grade bladder tumors among a sample of Iranian cases. Moreover, the *miR‐21* expression was noticeably increased in high‐grade tumors. There was significant correlation between the levels of *miR21* and *TGF‐β* expressions (Monfared et al., 2013). LncRNAs are nuclear or cytoplasmic noncoding RNAs involving in transcriptional or posttranscriptional gene regulation during cell proliferation and apoptosis (Enokida, Yoshino, Matsushita, & Nakagawa, 2016). Long intergenic noncoding RNAs (LincRNAs) are transcribed from intergenic regions which are associated with transcription and chromatin regulation (Vance & Ponting, 2014). *LINC00152* as a lincRNA has important regulatory roles in various cellular processes such as cell cycle, apoptosis, and cell migration (Lv et al., 2013; Szafranski et al., 2013). *LINC01082* (OMIM: 614978) can be also a transcriptional regulator of *FOXF1* (OMIM: 601089). It has been reported that there were decreased levels of *LINC00152* and *LINC01082* expressions in majority of a sample of Iranian BC tissues compared with their normal margins. The tumor type was significantly correlated with decreased levels of *LINC01082* expression. Moreover, they observed an association between age and *LINC00152* underexpression (Ousati Ashtiani, Pourmand, Salami, Ayati, & Tavakkoly‐Bazzaz, 2017). Exosomes are natural nanoparticles containing proteins and RNAs which are involved in urothelial tumorigenesis through microenvironment modification (Beckham et al., 2014). Exosomes exert their roles through LncRNAs transfer between cells (Hewson & Morris, 2016). The *MALAT1* (OMIM: 607924), *LINC00958* (OMIM: 618335), *LINC00355*, and *UCA1* (OMIM: 617500) are LncRNAs involving in regulation of cell proliferation, apoptosis, and migration (Han, Liu, Nie, Gui, & Cai, 2013; Seitz et al., 2017; Wang, Li, Xie, Zhao, & Chen, 2008). It has been observed that there were *MALAT1*, *LINC00355*, and *UCA1‐203* overexpressions in urinary exosomes of a sample of Iranian TCC cases compared with controls (Yazarlou, Modarressi, et al., 2018).

## 5 CELL SIGNALING AND KINASES

The PI3K/AKT/mTOR signaling is associated with cell growth, cell migration, and angiogenesis (McCubrey et al., 2012). Phosphatidylinositol 3‐kinases (PI3Ks) (OMIM: 601232) are lipid kinases producing PIP3 second messenger which induces cell growth and survival through AKT serine/threonine kinase (Cantley, 2002). The *mTOR* is also a serine/threonine kinase that is downstream mediator of PI3K/AKT pathway. It has been shown that the *PIK3CA* rs6443624 C>A and *AKT1* rs2498801 A>G were protective variants of BC. They observed that the rs1130233 G>A and rs2295080 G>T variants of *AKT1* (OMIM: 164730) and *mTOR*, respectively, increased the BC susceptibility among a subpopulation of Iranian patients (Bizhani et al., 2018). *PI3K* signaling pathway can be triggered by tyrosine kinase receptors (Knowles, Platt, Ross, & Hurst, 2009; Platt et al., 2009). *PIK3CA* mutational analysis was performed among a sample of Iranian BC patients which showed lower frequency of mutation in high‐grade and advanced stage tumors (Ousati Ashtiani, Mehrsai, Pourmand, & Pourmand, 2018). *HER‐2/neu* (OMIM: 164870) is a tyrosine kinase growth factor receptor. It has been observed that there was a correlation between higher grade of the TCC and *HER‐2/neu* overexpression is a sample of Iranian patients (Jalali Nadoushan, Taheri, Jouian, & Zaeri, 2007). Centrosomal protein 55 (*CEP55*) (OMIM: 610000) is an oncogene involving in cytokinesis and regulation of PI3K/AKT signaling pathway (Jeffery, Sinha, Srihari, Kalimutho, & Khanna, 2016). Forkhead box protein M1 (*FOXM1*) (OMIM: 602341) regulates expression of various genes such as CDC25B (OMIM: 116949), CCNB (OMIM: 123836), polo‐like kinase 1 (PLK1) (OMIM: 602098), and *CENP* (Laoukili, Stahl, & Medema, 2007). *PLK1* is also a serine/threonine kinase that induces cell proliferation through the cell‐cycle checkpoint skipping leading to genetic instability (He et al., 2011; Park, Sohn, Park, Chung, & Kim, 2011). It has been shown that there were upregulations of *CEP55*, *FOXM1*, and *PLK1* among a sample of Iranian BC tissues compared with their corresponding normal tissues (Seyedabadi et al., 2018). The fibroblast growth factor receptor (*FGFR*) as a tyrosine kinase receptor is one of the main regulators of cell‐cycle progression, angiogenesis, and embryonic development. It has been reported that there were *FGFR1* (OMIM: 136350) and *FGFR3* (OMIM: 134934) overexpressions in low‐grade and all tumors, respectively, among a subpopulation of Iranian BC subjects (Ousati Ashtiani, Tavakkoly‐Bazzaz, et al., 2017). The small G‐protein signaling modulator 3 (*SGSM3*) (OMIM: 610440) is a GTPase involving in regulation of G‐protein and Ras signaling (Yang, Sasaki, Minoshima, & Shimizu, 2007). It has been shown that the 4‐bp ins/del genotype and ins allele of *SGSM3* significantly increased the BC susceptibility among a subpopulation of Iranian patients (Hashemi et al., 2018). Sphingosine 1‐phosphate (*S1P*) (OMIM: 601965) is a bioactive lysosphingolipid mediator that binds to *S1PRn* (a G‐protein–coupled receptor) to regulate *MAPKs*. *S1PR3* is involved in tumor cell migration and self‐renewal. It has been reported that there were upregulation of *S1PR1*, *S1PR2*, and *S1PR3* expressions in tumor tissues compared with normal margins in a subpopulation of Iranian BC patients. There were also correlations among *S1PR1* overexpression, higher grades, and advanced stages (Palangi, Shakhssalim, Parvin, Bayat, & Allameh, 2019). *S100* (OMIM: 617427) are calcium‐binding proteins involving in cell proliferation, apoptosis, and inflammation. They are also the ligands for *RAGE* and Toll‐like receptors which activates the MAPK, PI3K‐AKT, and NF‐κB signaling pathways. *RAGE* (OMIM: 605762) as a receptor is associated with tumor progression and diabetes (Sessa et al., 2014; Xie, Mendez, Mendez‐Valenzuela, & Aguilar‐Hernandez, 2013). It has been reported that there were increased expressions of *S100A12* and *RAGE* in tumors in comparison with normal margins among a group of Iranian BC patients (Khorramdelazad et al., 2015). *IGFBP‐3* (OMIM: 146732) is the main carrier of IGF in blood stream. Moreover, *IGFBP‐3* affects cell signaling pathways and also binds with nuclear hormone receptors. It can be an oncogene or tumor suppressor based on tumor types. It has been observed that there were significant correlations between the A‐202C (rs2854744) polymorphism of *IGFBP‐3* and BC susceptibility in a sample of Iranian patients. The CC genotype carriers had more aggressive bladder tumors and the C allele was correlated with higher BC risk compared with 202‐A allele. Moreover, it was shown that the patient had significantly lower serum levels of *IGF‐1* (OMIM: 147440) and *IGFBP‐3* compared with controls (Safarinejad, Shafiei, & Safarinejad, 2011b). Tumor behavior and incidence rate can be associated with gender in which it has been observed that the aggressive tumors with poor prognosis are more frequent among females (Jemal, Siegel, Xu, & Ward, 2010; Miyamoto et al., 2012). This can be related to various factors such as different exposure to industrial chemicals, smoking, and hormonal differences (Bolenz, Lotan, Ashfaq, & Shariat, 2009; Mir et al., 2011). Sex steroid hormones function through their receptors in target cells (Miyamoto et al., 2012). It has been reported that there was a significant association between the AR expression and high‐grade and stage tumors in a sample of Iranian BC cases. AR‐positive cases had also significant higher rate of metastasis in comparison with AR‐negative cases. Therefore, they introduced AR as a prognostic marker among Iranian UBC (Mashhadi et al., 2014). Smoking and exposure to aromatic amines and polycyclic hydrocarbons are considered as the main environmental risk factors of BC (Cohen, Shirai, & Steineck, 2000). Jun‐terminal kinases (JNKs) are stress kinases activated following the ROS/RNS, heat, or osmotic shock which regulate cell cycle, DNA repair, and apoptosis through activation of several transcription factors such as *c‐JUN*, *ATF2* (OMIM: 123811), *P53*, and *ELK‐1* (OMIM: 311040) (Davis, 2000). Glutathione‐S‐transferases (GSTs) are involved in detoxification of xenobiotics, reactive oxygen species, and carcinogenic compounds (Schnakenberg, Breuer, Werdin, Dreikorn, & Schloot, 2000). Glutathione‐S‐transferase (GST) is a negative regulator switch for JNK. Low JNK activity is preserved through a GSTP1‐JNK complex in nonstressed cells. ROS or drugs dissociate the *GSTP1* (OMIM: 134660) from GSTP1‐JNK complex which activates the JNK and its subsequent downstream events (Yin, Ivanov, Habelhah, Tew, & Ronai, 2000). It has been reported that there were significant correlations between *GSTP1* Ile/Val or Val/Val genotypes and risk of BC in a group of Iranian patients. The *GSTP1* Val/Val genotype was significantly correlated with grade and stage of tumor. Due to a synergistic relationship among *GSTM1* (OMIM: 138350), *GSTT1* (OMIM: 600436), and *GSTP1*, the presence of *GSTM1* or *GSTT1* nulls with *GSTP1* Ile/Val or Val/Val genotypes significantly increased the BC susceptibility in this population. Moreover, the *GSTP1* Ile/Val genotype was protective against high‐grade and stage tumors (Safarinejad, Safarinejad, Shafiei, & Safarinejad, 2013).

## 6 CANCER TESTIS ANTIGENS AND EMT

Cancer testis antigens (CTAs) are a group of proteins mainly expressed in normal testis and tumor tissues which are associated with cell proliferation and epithelial–mesenchymal transition (EMT) (Forghanifard et al., 2011; Yang, Huo, Liao, & Zhou, 2015). Exosomes are extracellular nanoparticles normally can be detected in biofluids. Exosome contents can be assessed to identify the pathophysiology of related tissues. Such contents may involve in pathogenesis of various disorders via modification of intercellular communication and microenvironment (Sun, Luo, Jiang, & Duan, 2018; Valadi et al., 2007). A group have been assessed the expression of CTAs in urinary exosomes of a subpopulation of Iranian BC cases compared with controls. It has been observed that there was *MAGE‐B4* (OMIM: 300152) overexpression in TCC patients in comparison with controls. Moreover, the TCC cases had also higher levels of exosomal *NMP22* expression compared with BPH cases (Yazarlou, Mowla, et al., 2018). EMT is a critical process during the tumor progression and metastasis. E‐Cadherin as a cell adhesion protein is required for the normal integrity of epithelial cells and EMT process. Therefore, loss of *CDH1* expression results in increased cell migration and invasiveness in solid tumors. It has been reported that there was a correlation between *CDH1* expression and recurrent BC in a sample of Iranian patients (Khorrami et al., 2012). EMT is associated with various transcriptional repressors such *ZEB1* and Snail. *TGF‐β* upregulates the *ZEB1* expression via inhibition of *miR‐141*, *miR‐200a*, *miR‐200b*, and *miR‐200c*. It has been reported that the BC tissues had increased levels *ZEB1* mRNA expression compared with control tissues among a sample of Iranian cases. Moreover, there was a correlation between *TGF‐β2* overexpression and muscle invasion (Mahdavinezhad et al., 2017).

## 7 IMMUNE RESPONSE

Immune system is an important defense mechanism against the tumor cells (Smyth, Dunn, & Schreiber, 2006), however, the inflammation can be also an oncogenic mechanism through DNA damage and angiogenesis (Rakoff‐Nahoum, 2006). *IL‐6* (OMIM: 147620) is an inflammatory cytokine which is involved in immune response, cell proliferation, and apoptosis (Kishimoto, 2005). It has a key role in neoplastic transformation through *STAT3* (OMIM: 102582) (Iliopoulos, Jaeger, Hirsch, Bulyk, & Struhl, 2010). *IL‐12* (OMIM: 161,560) is another cytokine secreted by activated macrophages and dendritic cells (Colombo & Trinchieri, 2002) which inhibits tumorigenesis and angiogenesis (Sangro, Melero, Qian, & Prieto, 2005). It has been observed that there were significant correlations between *IL‐12* (3′UTR A>C) and *IL‐6* (‐174 C>G) genotypes and increased BC susceptibility among a subpopulation of Iranian cases. The GC and GC+CC genotypes carriers at C‐174G had higher risk of BC compared with GG genotype. In the case of *IL‐12*, there was a significant correlation between BC risk and AC genotype of *IL‐12B* (Ebadi et al., 2014). Chronic inflammation is one of the most important physiological processes involving in tumorigenesis. CD4+ T cells are categorized into T‐helper 1 (Th1) and Th2 (Murphy & Reiner, 2002) that are involved in cell‐mediated and humoral immune responses, respectively. Th17 cells are a class of CD4+ T cells producing *IL‐17A* (OMIM: 603149) inflammatory cytokine which regulates the physiological state of various immune and nonimmune cells. *IL‐17* induces the inflammation through proinflammatory cytokines and metalloproteinases. It has been reported that there was increased *IL‐17A* levels among a sample of Iranian BC cases compared with controls. Moreover, the advanced stage tumors had lower levels of *IL‐17A* compared with early stages tumors (Doroudchi et al., 2013). The Th17 cell differentiation is regulated by various factors such as *IL‐21*, *TGF‐β*, and *TNF‐α* (Nam et al., 2008; Shime et al., 2008). Th17 cells are associated with tumor regression through *IL‐17*, *VEGF*, and *TGF‐β* (Honorati, Neri, Cattini, & Facchini, 2006; Jeon et al., 2007). It has been shown that there were decreased serum levels of *IL‐17* and *TGF‐β* in a subpopulation of Iranian BC cases compared with controls. Decreased serum levels of *IL‐17* and *TGF‐β* were mainly in the early tumor stages which can be related to the inhibitory role of chemoradiotherapy on Th17 cells and TGF‐β‐producing tumor cells. They introduced *IL‐17* and *TGF‐β* as targets for the immunotherapy of advanced stage BC (Baharlou, Ahmadi Vasmehjani, Dehghani, Ghobadifar, & Khoubyari, 2014). Another study has been observed that there were increased and decreased levels of *IL‐17* and *TGF‐β,* respectively, in a sample of Iranian BC subjects. The *IL‐17* upregulation was observed in BC with early stage tumors. They concluded that the *IL‐17* probably decreased *TGF‐β* expressions in early stage of tumor progression (Baharlou et al., 2015).

## 8 CONCLUSIONS

In present review we summarized all of the reported genes until now among Iranian BC patients. To clarify the genetic and molecular biology of BC for the first time among Iranians, we categorized all of the significant reported genes based on their cell and molecular functions. Interestingly, we observed that the noncoding RNAs and epigenome are the main molecular processes associated with BC progression among Iranians. This review paves the way for determination of a population‐based panel of genetic markers for the early detection of BC among Iranians.

## CONFLICT OF INTEREST

The authors declare no conflicts of interest.

## AUTHOR CONTRIBUTIONS

Majid Mojarrad was involved in search strategy and drafting. Meysam Moghbeli designed the project and also was involved in edition and drafting. All authors read and approved the final manuscript.
